# miR-1307-5p suppresses proliferation and tumorigenesis of bladder cancer via targeting MDM4 and the Hippo signaling pathway

**DOI:** 10.1007/s12672-022-00512-2

**Published:** 2022-07-01

**Authors:** Wei Huang, Cheng Zhang, Shida Xiong, Xiaocheng Zhou, Gongxian Wang, Ju Guo

**Affiliations:** Department of Urology, The First Affiliated Hospital of Nanchang University, 17 Yongwaizheng street, Donghu District, Nanchang, 330006 Jiangxi China

**Keywords:** miR-1307-5p, MDM4, Hippo pathway, Bladder cancer

## Abstract

**Background:**

Emerging evidence has shown that miR-1307-5p is involved in tumorigenesis of various types of cancer. This study aims to assess the role and mechanism of miR-1307-5p in bladder cancer.

**Methods:**

Bioinformatics analyses were carried out with clinical datasets in the public domains. To investigate the cellular functions of miR-1307-5p, assays of cell proliferation, cell cycle and cell apoptosis were conducted in bladder cancer cell lines and xenografts. The molecular mechanisms of miR-1307-5p were studied using luciferase reporter, RT–qPCR, and western blotting analyses.

**Results:**

We found that miR-1307-5p expression was significantly decreased in bladder cancer tissues, and its lower level was associated with poor prognosis. Cellular assays indicated the tumor-suppressor roles of miR-1307-5p were linked to cell proliferation, cell cycle inhibition, and cell apoptosis promotion. Conversely, anti-miR-1307-5p facilitated cell proliferation and cell cycle and antagonized cell apoptosis. In the in vivo setting, tumor growth was suppressed by miR-1307-5p overexpression. We found by bioinformatic and luciferase reporter assays that miR-1307-5p targets the 3′-UTR of MDM4, a well-known Inhibitor of TP53-mediated transactivation, cell cycle arrest and apoptosis. Specifically, miR-1307-5p markedly reduced MDM4 proteins expression, decreased the expression of Ki-67 and PCNA, and increased the expression of cleaved-caspase 3 and caspase 9. While in parallel assays, anti-miR-1307-5p had opposite effects. In addition, we found that miR-1307-5p overexpression would suppress bladder cancer cell growth by inhibiting MDM4 and its downstream Hippo pathway.

**Conclusion:**

In bladder cancer, miR-1307-5p functions as a tumor suppressor and has the potentials as biomarker and therapeutical agent.

**Supplementary Information:**

The online version contains supplementary material available at 10.1007/s12672-022-00512-2.

## Introduction

As one of the most common genitourinary system malignancies, bladder cancer is characterized by high morbidity and mortality rates worldwide [1, 2]. In China, the incidence and mortality of bladder cancer showed an upward trend. There were roughly 80.5 per 100,000 newly diagnosed bladder cancer cases and 32.9 per 100,000 bladder cancer related deaths in 2015 [3, 4]. It is predicted that the incidence and mortality rate of bladder cancer would gradually increase yearly, imposing an enormous burden on public health [5, 6]. Clinically, the main treatment regimens for bladder cancer contain surgical resection and chemotherapy [7]. Although scientific advancements are improving the treatment of bladder cancer, the prognosis and the five-year survival rate of patients remains suboptimal [8]. Therefore, it is imminent to understand the molecular mechanisms of bladder cancer and explore novel biomarkers as potential diagnostic and therapeutic targets.

MicroRNAs (miRNAs) are a family of small noncoding RNAs about 22 nucleotides in length that are endogenously expressed [9]. By binding to the 3′-untranslated region (UTR) of target mRNAs, miRNA can negatively regulate the expression of target genes by inducing their degradation and translational repressions subsequently [10]. It has been documented that miRNA plays a critical role in a variety of tumor behaviors—including cell proliferation, apoptosis, drug resistance, and metastasis [11, 12]. Previous studies confirmed that miR-1, miR-133a, and miR-144-5p suppressed tumor growth and induced apoptosis, whereas miR-130b-3p and miR-556-3p promoted tumor development, migration, and invasion in bladder cancer. [13–16]

Aberrant expression of miR-1307 has been reported in several types of cancers. For example, in prostate cancer miR-1307 is tumorigenic via inhibiting FOXO3A signaling [17]. Biological pathways of miR-1307 cover cell differentiation and proliferation, metabolism, nucleotide synthesis, and lymphocytes activation [18]. However, miR-1307-5p expression and functions in bladder cancer remain undefined and in the present study, we aimed to systemically identify the cellular functions and molecular mechanisms of miR-1307-5p in bladder cancer in in vitro, in vivo, and clinical settings.

## Materials and methods

### Bioinformatic analysis

We retrieved the microarray expression of miR-1307-5p data from the GEO (Gene Expression Omnibus, https://www.ncbi.nlm.nih.gov/geo/) datasets and TCGA (The Cancer Genome Atlas, https://tcga-data.nci.nih.gov/tcga) database. GSE121711 datasets contains 10 primary bladder cancer tissues and 8 normal bladder tissues. The TCGA bladder cancer datasets included 429 bladder cancer samples after excluding cases with incomplete clinical information. The miR-1307-5p expression profiling in bladder cancer were analyzed using R statistical environment (R version 3.5.1). Patients were divided into high and low miR-1307-5p expression groups -using the median expression level—as the cut-off. The-Kaplan–Meier analysis was performed to for statistical significance in survival rates and generate the overall survival plots. P < 0.05 was set as—statistical significance.

### Clinical samples

A total of 20 pairs of primary bladder cancer tissues and matched noncancerous tissues were collected from bladder cancer patients, who received resection at The First Affiliated Hospital of Nanchang University in 2021. None of the patients received chemotherapy or radiotherapy prior to the surgery. The tumor pathological type was diagnosed by three independent pathologists, and the matched normal bladder epithelial tissues, which were collected from more than 5 cm away from the tumors, were simultaneously validated. The study was approved by the Ethics Committee of The First Affiliated Hospital of Nanchang University. Informed consent was obtained from each patient prior to their inclusion in the study.

### Cell culture and transfection

A normal human bladder uroepithelium cell line (SV-HUC-1) and bladder cancer cell lines (5637, T24, UMUC-3 and RT-4) used in this study were purchased from American Type Culture Collection (ATCC, Manassas, VA, USA) in August 2019. All cell lines were authenticated by short tandem repeat DNA profiling analysis in 2019. SV-HUC-1, 5637 and T24 cells were cultured in RPMI-1640 medium (Procell, Wuhan, China) supplemented with 10% fetal bovine serum and 1% double antibiotics (penicillin and streptomycin, NCM Biotech, China). UMUC-3 and RT-4 cells were cultured in DMEM (Procell, Wuhan, China) with 10% fetal bovine serum and 1% double antibiotics (penicillin and streptomycin, NCM Biotech, China). The cells were incubated in humidified air with 5% CO_2_ at 37℃. The miR-1307-5p mimics, miR-1307-5p inhibitor, small interference RNAs of MDM4 and their negative controls were obtained from Shanghai GenePharma Co, Ltd (Shanghai, China). Cell transfection was conducted using Lipofectamine 2000 (Invitrogen, Carlsbad, CA, USA). Upon 72 h of transfection, analyses were carried out as indicated.

### RNA extraction and real-time RT–qPCR

Total RNA was extracted from cells or tissue samples using TRIzol reagent (Thermo, USA). cDNA synthesis was carried out using the reverse transcription kit (Genecopoeia, Guangzhou, China). The sequence of specific miR-1307-5p reverse transcription primer was shown as follow: 5′ -GTCGTATCCAGTGCGTGTCGTGGAGTCGGCAATTGCACTGGATACGACAGCCGG-3′. We used BeyoFast™ SYBR Green PCR kit (Bio-Rad, Shanghai, China) for real-time qPCR, under the following conditions: 1× (− 95 ℃, 2 min) and 40× (95 ℃, 15 s; 60 ℃, 30 s). The primers for RT–qPCR analysis of miR-1307-5p were as follows: forward, 5′-TCGACCGGACCTCGA-3′, and reverse, 5′-CAGTGCGTGTCGTGGA-3′. MDM4 forward and reverse primers are: 5′-TGATTGTCGAAGAACCATTTCGG-3′ and 5′-TGCAGGGATCAAAAAGTTTGGAG-3′.—U6 small nuclear RNA or glyceraldehyde 3-phosphate dehydrogenase (GAPDH) was used as internal controls, respectively.

### Western blotting analysis

Cells were lysed with RIPA lysis buffer kit (Beyotime, Shanghai, China), and—protein samples were quantified using the BCA protein quantification kit (Beyotime Biotechnology, Shanghai, China). Total protein lysates (25 ug) were resolved by 10% sodium dodecyl sulfate–polyacrylamide gel electrophoresis (SDS-PAGE) and transferred to PVDF membranes. Membranes were blocked with 5% skim milk for 1 h at room temperature, followed by incubation for overnight at 4 ℃ with the primary antibodies: anti-GAPDH (1:6000, Proteintech, Beijing, China), anti-Ki-67 (1:1000, Proteintech, Beijing, China), anti-MDM4 (1:1500, Proteintech, Beijing, China), anti-PCNA (1:2000, Cell Signaling Technology, USA), anti-caspase9 (1:1000, CST, USA), anti-caspase3 (1:1000, CST, USA), anti-YAP (1:5000, Proteintech, Wuhan, China), and anti-LATS1 (1:2000, Proteintech, Wuhan, China). After washing with TBST buffer for three times, membranes were incubated with secondary antibody (1:10,000, CST, USA) for 1 h at 37 ℃. ECL was used for development and Biolmaging Systems was used for signals detection and visualization (see Tables 1, 2).

**Table 1:** Primer sequence

Genes	Sequence
miR-1307-5p RT primer	GTCGTATCCAGTGCGTGTCGTGGAGTCGGCAATTG CACTGGATACGACAGCCGG
miR-1307-5p Forward primer	TCGACCGGACCTCGA
miR-1307-5p Reverse primer	CAGTGCGTGTCGTGGA
MDM4 Forward primer	TGATTGTCGAAGAACCATTTCGG
MDM4 Reverse primer	TGCAGGGATCAAAAAGTTTGGAG
GAPDH Forward primer	ACAGCCTCAAGATCATCAGC
GAPDH Reverse primer	GGTCATGAGTCCTTCCACGAT
U6 Forward primer	CTCGCTTCGGCAGCACA
U6 Reverse primer	AACGCTTCACGAATTTGCGT

*MDM4* mouse double minute 4; *GAPDH* glyceraldehyde-3-phosphate dehydrogenase

**Table 2:** Antibodies

Names	Dilution	Item No.	Company
GAPDH	1: 6000	60,004-1-Ig	Proteintech, Beijing, China
Ki-67	1: 1000	27,309-1-AP	Proteintech, Beijing, China
MDM4	1: 1500	17,914-1-AP	Proteintech, Beijing, China
PCNA	1: 2000	2586	Cell Signaling Technology, Beverly, CA, USA
caspase9	1: 1000	9509	Cell Signaling Technology, Beverly, CA, USA
caspase3	1: 1000	9662	Cell Signaling Technology, Beverly, CA, USA
Anti-mouse IgG (H + L)	1: 10,000	14,709	Cell Signaling Technology, Beverly, CA, USA
YAP	1: 5000	13,584-1-AP	Proteintech, Wuhan, China
LATS1	1: 2000	17,049-1-AP	Proteintech, Wuhan, China

*MDM4* mouse double minute 4; *GAPDH* glyceraldehyde-3-phosphate dehydrogenase; *PCNA* proliferating cell nuclear antigen; *YAP* yes associated protein; *LATS1* large tumor suppressor kinase 1

### CCK-8 assay

Cell viability was measured using the Cell Counting Kit-8 assay kit (Sigma, USA). Cell suspensions were seeded in 96-well plates at a density of 3000 cells per-well with 3 repeats for each test. At 0, 24, 48, and 72 h after inoculation respectively, 100 ml CCK-8 was added to each well and incubated for additional 1 h at 37 ℃. The absorbance values were measured at 450 nm using a microplate reader.

### Cell cycle analysis

Cells were digested with trypsin/EDTA (NCM Biotech, China), and washed twice with cold PBS. Then, the collected cells were fixed in pre-cold 70% ethanol for 24 h at 4 ℃ and incubated with RNase A for 30 min. After that, the cells were stained with propidium iodide (PI, Beyotime, Shanghai, China) for 10 min in darkness. Finally, cell cycle was determined with flow cytometry (BD Biosciences) and the percentage of cells in different phases was analyzed using FlowJo 10 software.

### Cell apoptosis assay

Cell apoptosis was determined by Annexin V and PI double staining protocol using the Annexin V-FITC apoptosis detection kit (KeyGen Biotech, China). Cells were harvested with trypsin, washed with PBS, pelleted in a cooled centrifuge, and resuspended in binding buffer. Next, cells were incubated with Annexin V-FITC and propidium iodide (PI) and maintained in darkness for 15 min at room temperature. Flow cytometric analysis was performed with a FACS Calibur instrument (BD Biosciences). The results were analyzed by FlowJo 10 software.

### In vivo tumorigenicity* assay*

Twelve six-week-old male BALB/c nude mice were obtained from Shanghai Experimental Animal Center (Shanghai, China), which weighted about 18–20 g each. All mice were housed in a strict pathogen-free conditions. All animal experiments were performed using protocols approved by the Animal Center of The First Affiliated Hospital of Nanchang University. A random number table method was used to divide the mice into two groups, negative control group and miR-1307-5p mimics group (N = 6 for each group). Each nude mouse was subcutaneously inoculated with 100 µL of cell suspension (1 × 10^6^ cells) in the right axillary sub cutis. The length and width of the tumor was measured every 4 days using a vernier caliper, and the tumor size was calculated as follows: volume (mm^3^) = 0.5 × length (mm) × width^2^(mm^2^), and the growth curve was calculated. At the end point, the mice were euthanized, the tumors were collected and weighed. During the experiment, care was taken to minimize the pain of the mice without affecting the results of the experiment. No deaths occurred during the experiment.

### Luciferase reporter assay

The luciferase reporter assay was conducted for miRNA target validation. TargetScan (http://www.targetscan.org/vert_72/), a biological prediction website, predicted that 3′-UTR sequences of MDM4 might be the direct target of miR-1307-5p. In order to construct the luciferase plasmid, the MDM4 3′-UTR gene sequence or a mutant sequence were inserted into pGL3 promoter vector (Invitrogen, USA), which was defined as wt-MDM4-3′-UTR and mut-MDM4-3′-UTR. According to the manufacturer’s protocol, T24 cells that transfected with miR-1307-5p mimics or miR-NC (negative control) were co-transfected with luciferase reporter plasmid and Renilla luciferase (internal control) vector using Lipofectamine 2000 (Invitrogen, USA). While 5637 cells that transfected with miR-1307-5p inhibitor or miR-NC were co-transfected with luciferase reporter plasmid and Renilla luciferase control vector using Lipofectamine 2000 (Invitrogen, USA). Cells were collected and lysed after 48 h transfection, followed by analyses using the Dual Luciferase Reporter Assay Kit (Promega, USA); reporter activity was calculated based on firefly luciferase—normalized to Renilla luciferase.

### Statistical analyses

Data were expressed as the mean ± standard deviation (SD). Differences between two independent groups were analyzed using a two-tailed Students’ *t*-test. Differences between multiple groups were analyzed using ANOVA tests. Survival curves were analyzed using the Kaplan–Meier method and compared using the log-rank test. A p-value < 0.05 was considered statistically significant.

## Results

### miR-1307-5p downregulation is correlated with poor bladder clinical outcomes

**Fig. 1:**
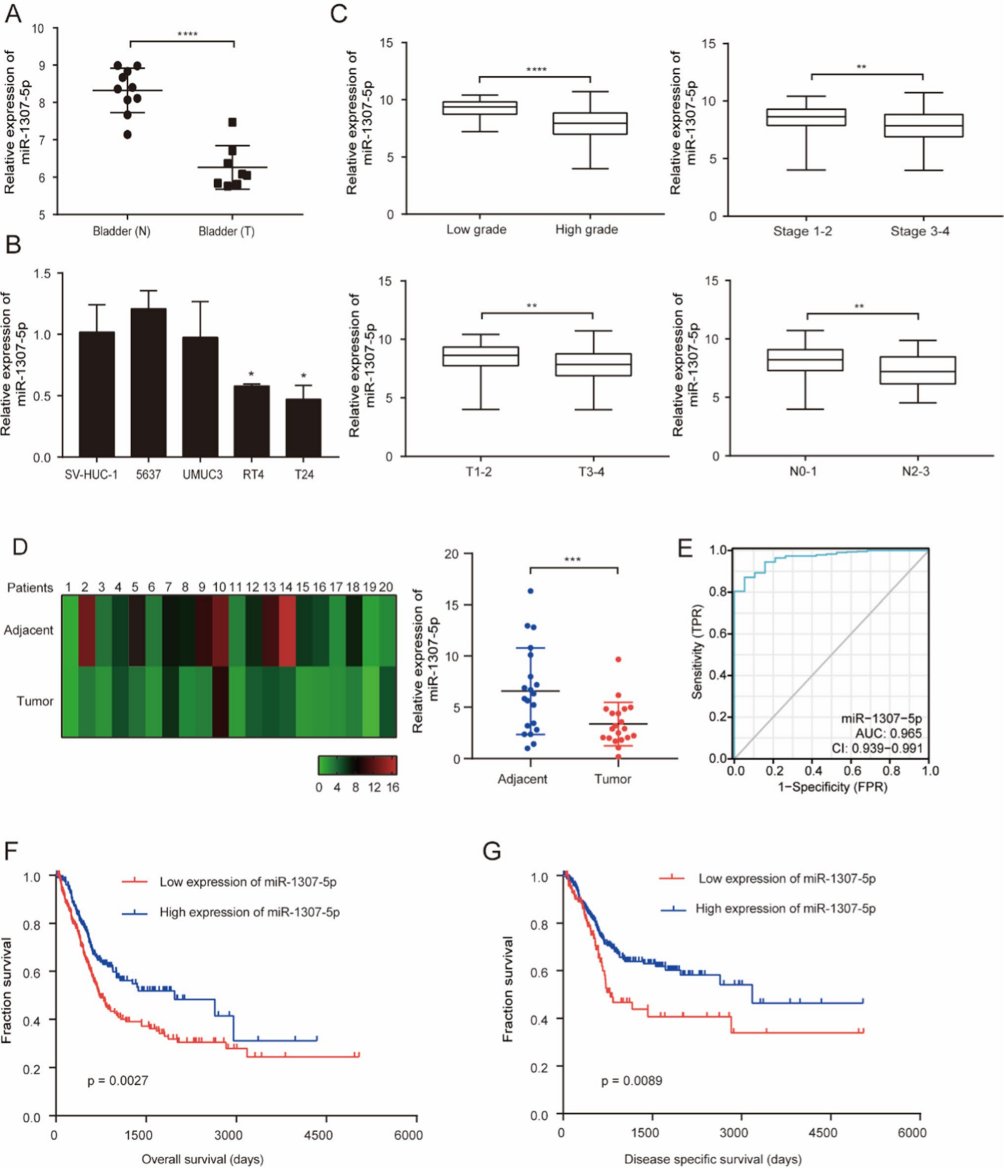
Inverse correlation between miR-1307-5p expression and—bladder cancer clinical grades. **A** Expression levels of miR-1307-5p in clinical bladder cancer tissues versus normal bladder tissues, based on analysis of the GEO datasets. **B** Expression levels of miR-1307-5p in bladder cancer cell lines and a normal human bladder uroepithelium cell line was determined by RT–qPCR. **C** Loss of miR-1307-5p coincide with worse clinicopathological features, as analysis of the TCGA datasets. **D** miR-1307-5p expression in 20 paired bladder tumor tissues versus adjacent normal tissues. **E** RT–qPCR analysis—of miR-1307-5p in tumor tissues and matched normal tissues. **F** Kaplan–Meier’s analysis of clinical cases based on miR-1307-5p expression and overall survival rates. **G** Kaplan–Meier’s analysis of clinical cases based on miR-1307-5p expression and disease specific survival rates. *p < 0.05; **p < 0.01; ***p < 0.001; ****p < 0.0001. Data depicts the mean ± standard deviation and are representative of three independent experiments. TNM means tumor node metastasis. Low grade means part of grade 1 carcinomas and part of grade 2 carcinomas, High grade means part of grade 2 carcinomas and grade 3 carcinomas

To investigate the clinical impacts of miR-1307-5p on bladder cancer, we downloaded and analyzed miRNA expression datasets of bladder cancer tissues and normal bladder tissues from GEO database (GSE 121,711). As shown in Fig. 1A, miR-1307-5p expression in bladder cancer tissues was significantly lower than that in normal tissues (P < 0.0001). The expression of miR-1307-5p was next monitored by RT–qPCR in bladder cancer cell lines (5637, UMUC3, RT4, T24) and the normal human bladder uroepithelium cell line (SV-HUC-1) (Fig. 1B). miR-1307-5p was significantly lower in bladder cancer patients with high grade (advanced tumor stage III–IV) than those with low grade (early tumor stage I–II) (Fig. 1C). miR-1307-5p levels in bladder cancer patients with T1–T2 and N0-N1 were significantly higher than those in cases with T3-T4 and N2-N3, respectively (Fig. 1C). In order to validate miR-1307-5p expression profile in clinical settings, total RNA from 20 bladder cancer tissues and matched noncancerous bladder tissues obtained from patients cohort was assessed using real-time RT–qPCR analysis. Our data showed that the expression of miR-1307-5p in bladder cancer tissues was significantly lower than that in tumor-adjacent normal tissues (Fig. 1D). From the ROC curve of diagnosis, miR-1307-5p expression showed an ability to accurately identify tumor from normal tissue (Fig. 1E, AUC = 0.965). Moreover, the survival analyses of TCGA database indicated that bladder cancer patients with low miR-1307-5p expression had a significantly poorer overall survival and disease specific survival rate compared to those with high miR-1307-5p expression (Fig. 1F, G). Hence, these findings are consistent with miR-1307-5p being a tumor-suppressor in bladder cancer.

### *miR-1307-5p inhibits proliferation of bladder cancer cells *in vitro

**Fig. 2:**
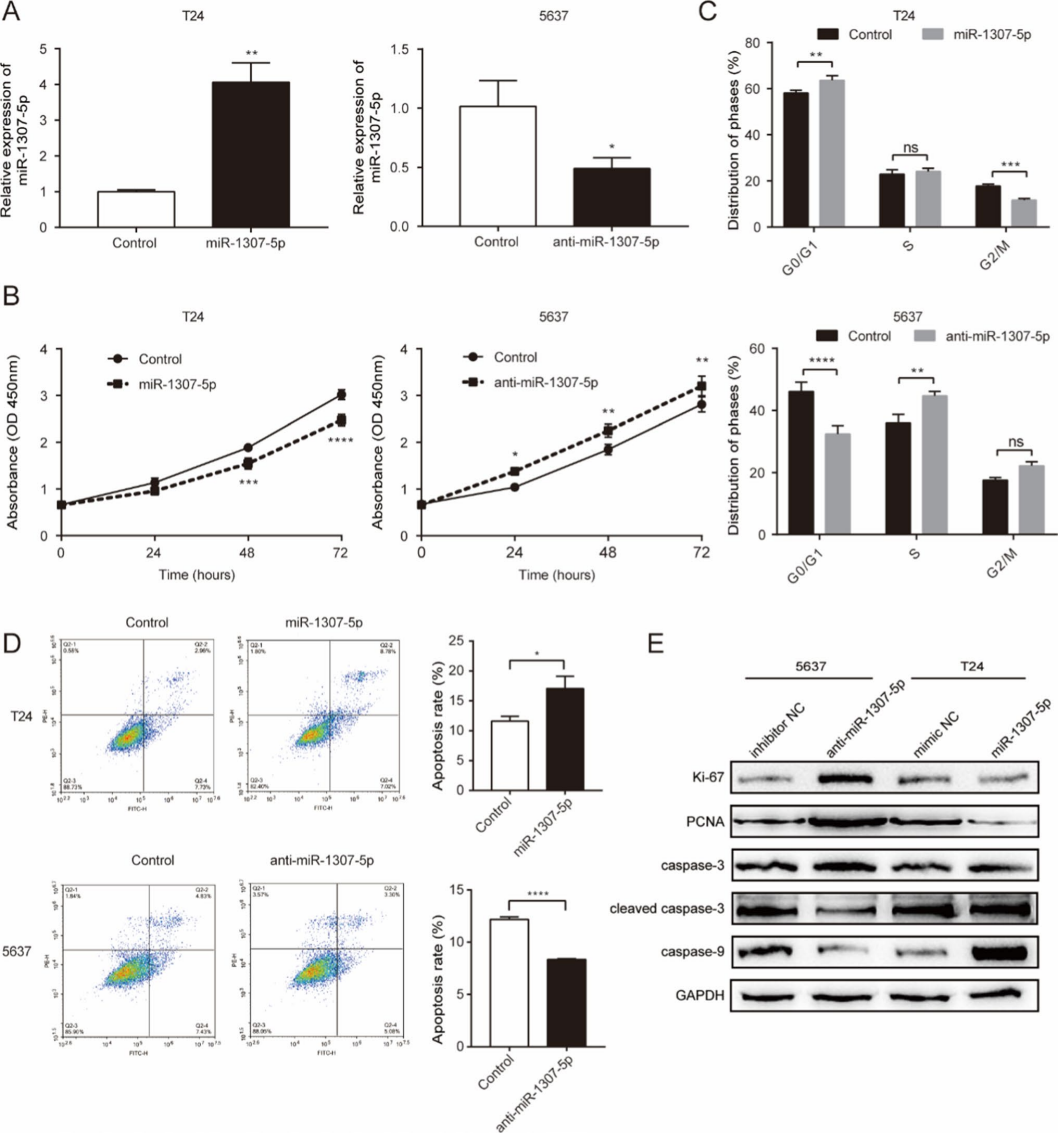
miR-1307-5p inhibits cell proliferation and cell cycle progression and elicits cell apoptosis of bladder cancer cells in vitro. **A** Expression of miR-1307-5p in T24 and 5637 cells that were transfected with indicated vectors was determined by RT–qPCR. **B** CCK-8 assay was performed on cell proliferation upon transfection with miR-1307-5p mimics or inhibitor. **C** Flow cytometry assay with PI staining was performed on cell cycle. **D** Flow cytometry assay with Annexin V and PI staining was performed on cell apoptosis. **E** Expression of proliferation and apoptosis markers following miR-1307-5p stable overexpression or inhibition. *p < 0.05; **p < 0.01; ***p < 0.001; ****p < 0.0001; ns, no significance. Data depicts the mean ± standard deviation and are representative of three independent experiments

To explore the cellular functions of miR-1307-5p in bladder cancer, overexpression versus knock-down tests were performed in T24 versus 5637 cell lines respectively, which had different levels of endogenous miR-1307-5p. Based on the results of RT–qPCR assay, transfection was successful for both miR-1307-5p mimics overexpression in T24 cells and miR-1307-5p inhibition in 5637 cells (Fig. 2A). CCK-8 assay was conducted to assess the effect of miR-1307-5p on cell proliferation; and the results indicated -that inhibition of miR-1307-5p in 5637 cells increased cell proliferation while upregulation of miR-1307-5p in T24 cells inhibited cell proliferation (Fig. 2B). In accordance, the expression of proliferation markers (Ki-67 and PCNA) increased in 5637 cells upon miR-1307-5p inhibition but decreased in T24 cells upon miR-1307-5p overexpression (Fig. 2E). These results suggested miR-1307-5p inhibits bladder cancer cell proliferation.

### miR-1307-5p elicits cell cycle arrest in the G0/G1 phase in vitro

To determine the underlying mechanisms of miR-1307-5p cell growth inhibitory effects in bladder cancer cells, we assessed using flow cytometry the cell cycle of T24 cells transfected with miR-1307-5p mimics and 5637 cells transfected with miR-1307-5p inhibitor. Compared with the control group, miR-1307-5p overexpression in T24 cells increased the G0/G1 phase cell populations and decreased the proportion of G2/M phase cells (Fig. 2C). In contrast, miR-1307-5p inhibition in 5637 cells substantially reduced the fraction of G0/G1 phase cells and enhanced the abundance of S phase cells (Fig. 2C). Taken together, these results suggested one mechanism of miR-1307-5p anti-proliferation functions is mediated by G0/G1 cell cycle arrest.

### miR-1307-5p promotes the apoptosis of bladder cancer cells in vitro

Flow cytometric assay with Annexin V and PI staining was then performed to assess cell apoptosis accordingly. The results suggested that the apoptosis rates in T24 cells overexpressed of miR-1307-5p mimics and the control groups were 17.1 ± 2.0 and 11.6 ± 0.8, respectively (Fig. 2D). In comparison, we observed in 5637 cells transfection of miR-1307-5p inhibitor markedly reduced cell apoptosis (Fig. 2D). Next, we examined the expression level of typical apoptotic effectors (caspase3, cleaved caspase3 and caspase9) and found that cleaved caspase3 and caspase9 were higher in T24 cell overexpressed of miR-1307-5p mimics than that in control cells. In contrast, in 5637 cells transfected of miR-1307-5p inhibitor the expression of cleaved caspase3 and caspase9 were much lower than that in control cells (Fig. 2E). Thus, we determined miR-1307-5p elicited apoptosis of bladder cancer cells.

### miR-1307-5p inhibits tumorigenesis of bladder cancer cells in vivo

**Fig. 3:**
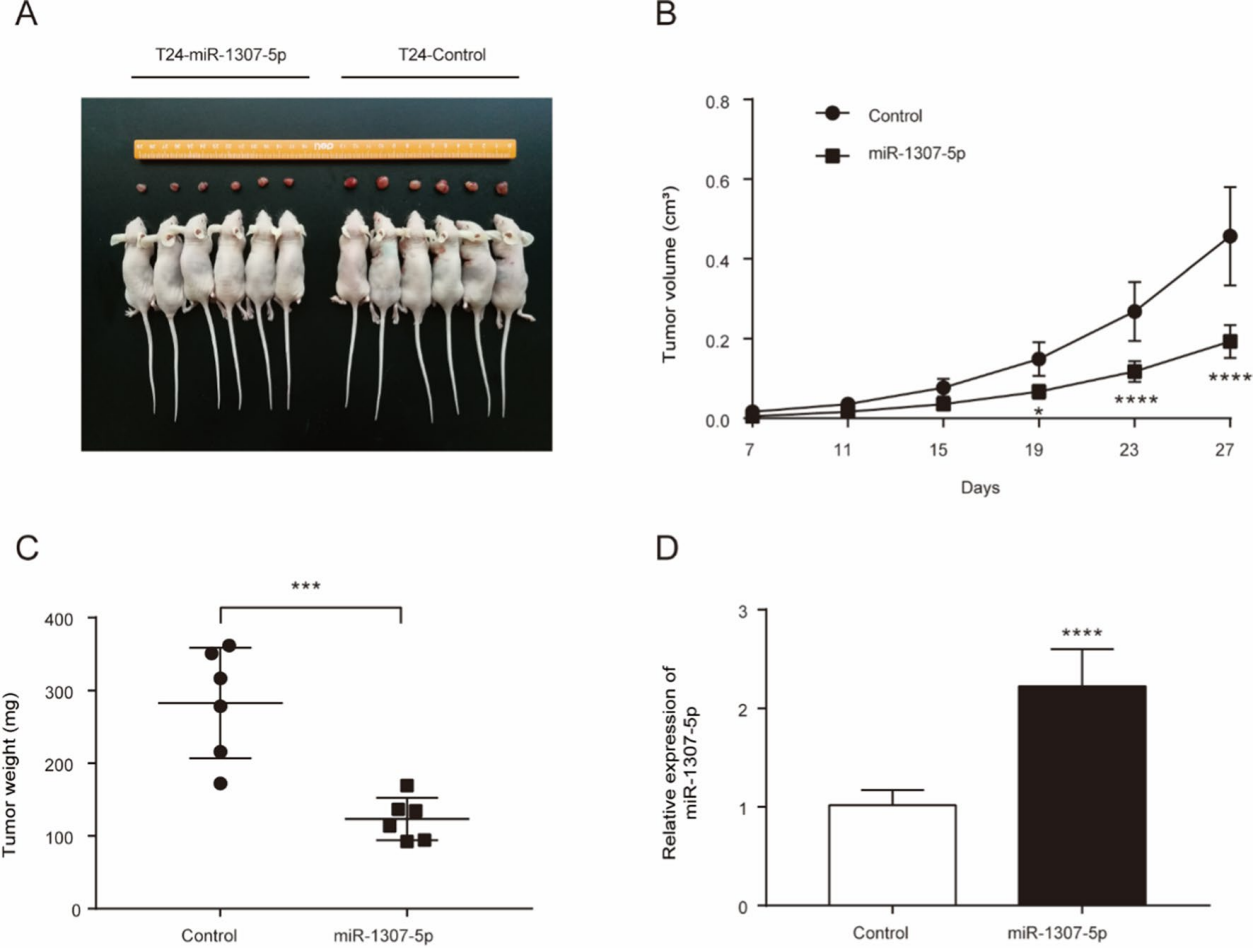
miR-1307-5p inhibits bladder cancer growth in a xenograft nude mice model. **A** Macrograph of tumors in all groups. **B** Calculated tumor volume. **C** Calculated tumor weight. **D** Expression of miR-1307-5p as determined by RT–qPCR. *p < 0.05; ***p < 0.001; ****p < 0.0001. Data depicts the mean ± standard deviation and are representative of three independent experiments

To further verify the antitumor effect of miR-1307-5p in bladder cancer, xenograft tumor model was generated by injecting LV with control vector versus miR-1307-5p mimics expression plasmid into the left axillary sub cutis of nude mice, respectively. As shown in Fig. 3A, transfection of miR-1307-5p mimics significantly inhibited tumor growth (as evidenced in tumor volume, weight and tumor growth curves) in nude mice compared to the control group- (Fig. 3B and C). RT–qPCR confirmed that miR-1307-5p was significantly increased in the miR-1307-5p overexpression group compared to the control group (Fig. 3D). These findings indicated the bladder cancer suppressive functions of miR-1307-5p in the in vivo settings.

### miR-1307-5p targets MDM4 to modulate cell growth in bladder cancer cells

**Fig. 4:**
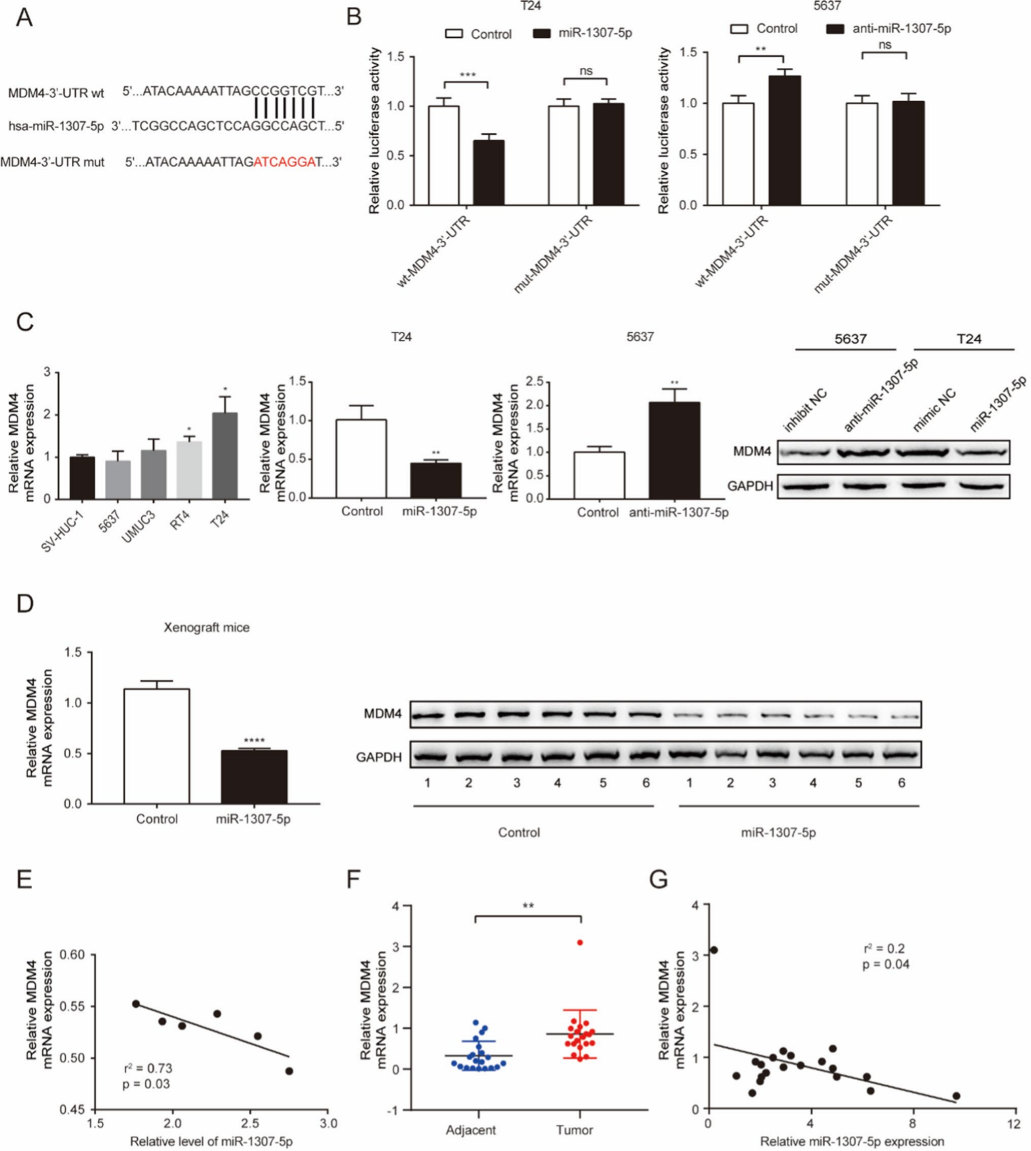
MDM4 is a direct target of miR-1307-5p in bladder cancer cells. **A** Diagrams showing the putative miR-1307-5p binding site of MDM4 locus and corresponding mutant sites. **B** miR-1307-5p effects on luciferase reporters based on wild-type (wt) MDM4 3′-UTR versus mutant (mt) 3′-UTR. **C** T24 and 5637 cells that were transfected with corresponding miRNA vectors were subjected to RT–qPCR and western blotting for MDM4 expression. **D** RT–qPCR and western blotting analyses to compare MDM4 expression in xenografts. **E** An inverse correlation between miR-1307-5p and MDM4 mRNA expression in vivo. **F** RT–qPCR analysis of MDM4 in tumor tissues and matched normal tissues. **G** Inverse correlation between miR-1307-5p and MDM4 expression in -Spearman’s rank correlation analysis. **p < 0.01; ****p < 0.0001; ns, no significance. Data depicts the mean ± standard deviation and are representative of three independent experiments

To further investigate the molecular mechanism of miR-1307-5p inhibitory effects on bladder cancer cell growth, we then focused on its potential direct target gene. We used publicly available algorithms TargetScan to predict the targets of miR-1307-5p, and a sequence complementary to the 3′-UTR of MDM4 mRNA was identified. To test the binding specificity between miR-1307-5p and MDM4 3′-UTR, we developed a dual luciferase reporter system. As shown in Fig. 4A, firefly reporters driven by the miR-1307-5p complementary wild type (wt) binding site (wt-MDM4-3′-UTR) versus the mutant binding site (mut-MDM4-3′-UTR) was constructed. Co-transfection T24 cells of wt-MDM4-3′-UTR reporter and miR-1307-5p mimics significantly decreased luciferase activity compared with the control. However, co-transfection of mut-MDM4-3′-UTR reporter and miR-1307-5p mimics did not change the luciferase activity. Moreover, co-transfection 5637 cells of wt-MDM4-3’-UTR plasmid and miR-1307-5p inhibitor markedly increased luciferase activity compared with the control. In contrast, co-transfection of mut-MDM4-3′-UTR plasmid and miR-1307-5p inhibitor did not change the luciferase activity (Fig. 4B). Furthermore, RT–qPCR and western blotting analyses showed that endogenous MDM4 mRNA and protein levels in T24 cells were decreased upon miR-1307-5p overexpression compared with the control cells. On contrary, miR-1307-5p knockdown in 5637 cells substantially increased MDM4 expression (Fig. 4C). Taken together, these findings indicated MDM4 is endogenous target of miR-1307-5p. Importantly, MDM4 expression was markedly decreased upon miR-1307-5p overexpression and negatively correlated with miR-1307-5p expression in the mouse tumor tissues (Fig. 4D, E). To validate these observations in clinical settings, we next analyzed the messages of 20 paired patient tissues and found that MDM4 mRNA was significantly upregulated in bladder cancer tissues compared with their adjacent normal tissues (P = 0.01, Fig. 4F). Indeed, a significant inverse correlation existed in bladder cancer tissues between the RNA levels of miR-1307-5p and MDM4, as evaluated by Spearman’s correlation test (Fig. 4G). Collectively, the results indicated in in vitro, in vivo, and clinical settings that MDM4 is a direct downstream target of miR-1307-5p in bladder cancer.

### MDM4 and the Hippo pathways are key mediators of miR-1307-5p effects in bladder cancer

**Fig. 5:**
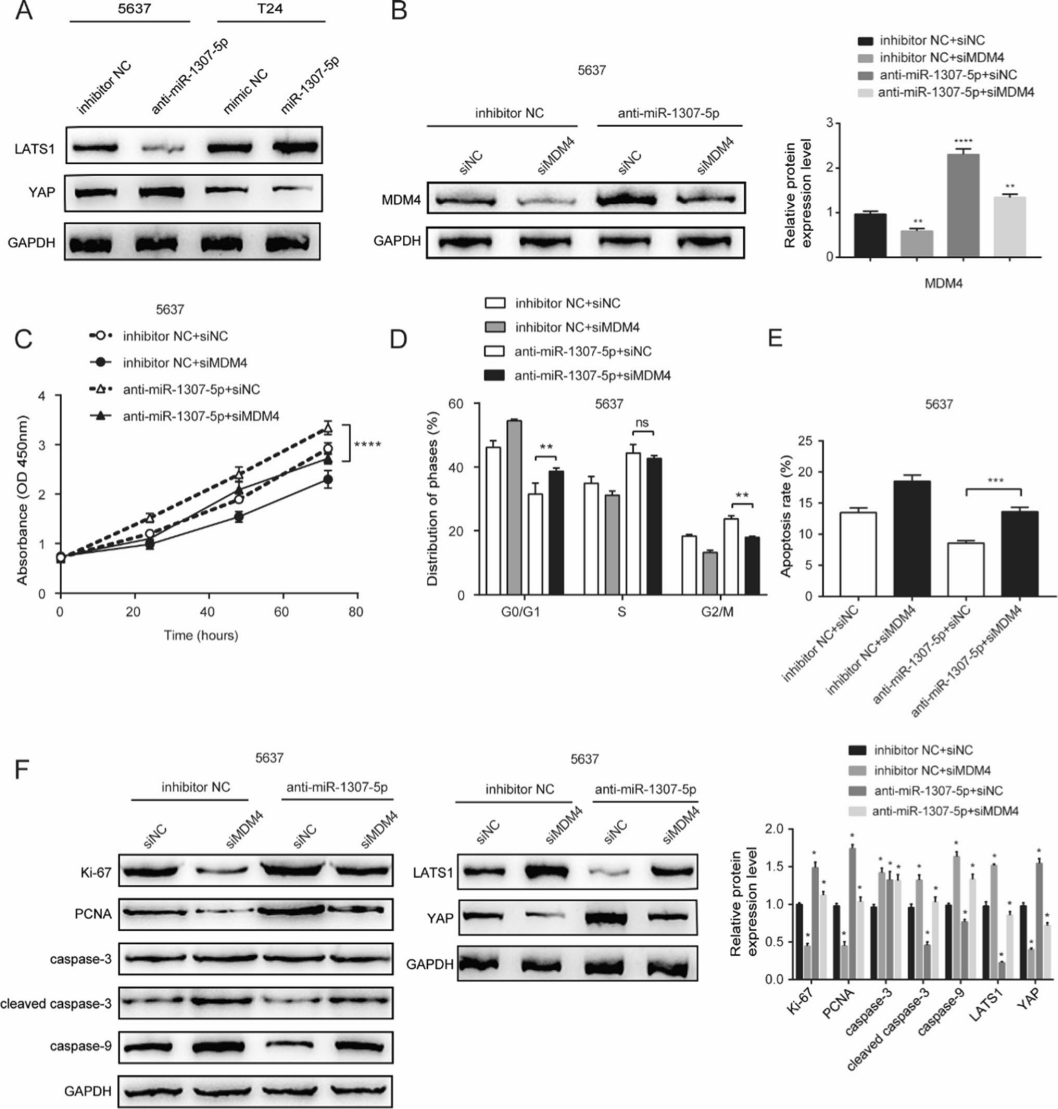
miR-1307-5p effects were mediated by—MDM4 and the Hippo pathway. **A** Western blotting of LATS1 and YAP in 5637 and T24 cells. **B** Western blotting of MDM4 protein in 5637-anti-miR-1307-5p cells—transfected with siMDM4. **C** Proliferation of 5637-anti-miR-1307-5p cells transfected with siMDM4 as determined by CCK-8 assay. **D** Cell cycle of 5637-anti-miR-1307-5p cells transfected with siMDM4 as determined by flow cytometry assay with PI staining. **E** Flow cytometry assay with Annexin V and PI staining—to determine the effects of MDM4 on the cell apoptosis of bladder cancer cells. **F** MDM4 knockdown decreased the expression of Ki-67, PCNA, YAP and increased the levels of cleaved caspase3, caspase9, LATS1 in 5637-anti-miR-1307-5p cells. **p < 0.01; ***p < 0.001; ****p < 0.0001; ns, no significance. Data depicts the mean ± standard deviation and are representative of three independent experiments.

Our GO and KEGG enrichment analysis of miR-1307-5p and MDM4 associated genes indicated that miR-1307-5p and MDM4 were significantly related to Hippo signaling pathway (Figure S1A and S1B). As shown in Fig. 5A, we found that LATS1 expression was changed similarly to miR-1307-5p upon miR-1307-5p knockdown or overexpression. Meanwhile, YAP expression was inversely altered by miR-1307-5p knock-down or overexpression. Next, rescue experiments were performed to validate whether miR-1307-5p executed its functional effects by through its targets. Figure 5B showed in 5637 cells that compared with miR-1307-5p inhibitor transfection, MDM4 proteins decreased upon co-transfection of miR-1307-5p inhibitor and siMDM4. Functionally, increased cell viability and cell cycle progression and decreased cell apoptosis caused by miR-1307-5p downregulation were partially abolished upon MDM4 silence, as determined by CCK-8 and flow cytometry analyses (Fig. 5C–E), respectively. Furthermore, as shown in Fig. 5F, Ki-67 and PCNA expression was upregulated, while cleaved caspase3 and caspase9 were downregulated upon transfection of miR-1307-5p inhibitor. In comparison, MDM4 silence-in miR-1307-5p inhibited 5637 cells led to a pronounced reduction of Ki-67 and PCNA and enhancement of cleaved caspase3 and caspase9, as compared with the miR-1307-5p inhibitor group. At cellular levels, MDM4 silence in 5637 cells would reverse the pro-proliferative and anti-apoptotic effects of miR-1307-5p inhibitor. Notably, Western blotting illustrated that the LATS1 proteins were enhanced while YAP proteins were reduced in 5637 cells upon co-transfection of miR-1307-5p inhibitor and si-MDM4 (Fig. 5F). MDM4 silence again reversed the effects of miR-1307-5p inhibitor on the expression of LATS1 and YAP. These results indicated that miR-1307-5p inhibited tumorigenesis by directly targeting MDM4 3′UTR to down-regulate its expression, thereby inhibiting the Hippo pathway in bladder cancer.

## Discussion

Bladder cancer is the ninth most common form of cancer and ranks 13th in terms of yearly mortality from cancer worldwide [19]. Nearly three-fourths of patients experienced recurrence after transurethral resection [20, 21]. The molecular mechanisms of bladder cancer on-set and progression are largely undefined. Therefore, it is imperative dissect relevant prognostic and therapeutic targets in bladder cancer.

miRNAs function in numerous cellular biological processes including cell proliferation, differentiation, apoptosis, stress response, and development [22]. The expression profiles of miRNAs have been applied to diagnosis and prognosis of many human malignancies [23]. Cellular analyses revealed that miR-1307-5p ectopic overexpression versus inhibition had opposite effects on cell proliferation, cell cycle progression, and cell apoptosis in bladder cancer cell lines. Of significance, the cellular effects of miR-1307-5p were reproduced in bladder cancer xenografts in vivo.

It is well known that molecules play different roles, even totally opposite roles, in different cells or tissues [24]. In our study, miR-1307-5p has been found as an antioncogene in bladder cancer. But it is interesting to note that the role of miR-1307 in different tumors is not the same and sometimes even the opposite. Qiu et al. reported that miR-1307 was highly expressed in prostate cancer and contributed to prostate cancer proliferation by targeting FOXO3A [17]. But Zheng et al. found miR-1307 was decreased in colon cancer and inhibited proliferation and promote apoptosis through targeting downregulation of ISM1 [25]. These studies have elucidated that the function of miR-1307 is very complex and varies in different cancers. The reason miR-1307 plays opposite functions in different tumor cells may be due to the different targets it is binding to. And research indicated that miRNA could regulate a variety of mRNAs after the transfection. The roles these genes played in different tissues or tumors are different, which may be the reason for the diversity of miRNA functions [26].

By bioinformatics screening, we identified MDM4 3′UTR is sequence complementary to miR-1307-5p. Followed reporter and endogenous gene expression analyses implied MDM4 as a direct miR-1307-5p down-stream target. Rescue tests validated that the cellular effects of miR-1307-5p were partially mediated by MDM4.

MDM4 belongs to a family of the mouse double minute oncoproteins, known for its dynamic negative regulation of the major tumor suppressor p53 [27]. MDM4 contributes to p53 inhibition by suppressing its transcriptional activity, and also by partnering with MDM2 to regulate p53 degradation [28]. Stegeman et al. reported that increased MDM4 expression is associated with prostate cancer metastasis and recurrence [29]. Evidences consistently suggested that MDM4 is overexpressed in various types of human cancer and are highly associated with tumorigenic processes including cell apoptosis, proliferation, metastasis, invasion, as well as the resistance to chemotherapy [30]. We further found miR-1307-5p significantly inhibited the Hippo pathway via MDM4 in bladder cancer. Taken together, our findings indicated the tumor suppressor functions of miR-1307-5p in bladder cancer and its anti-tumor effectors include MDM4 and the Hippo pathway.

## Conclusions

In conclusion, miR-1307-5p overexpression could suppress tumorigenesis of bladder cancer by inhibiting MDM4 and its downstream Hippo pathway in bladder cancer. Hence, miR-1307-5p has the potentials as biomarker and therapeutical agent in bladder cancer.

## Supplementary Information

Additional file 1: Figure S1A. Bubble plot of miR-1307-5p from the GO enrichment analysis. (PDF 8 KB)

Additional file 2: Figure S1B. Bubble plot of miR-1307-5p from the KEGG enrichment analysis (PDF 8 KB)

## Data Availability

The datasets generated during or analyzed during the current study are available from the corresponding author on reasonable request.
