# Supplementary figures and images for: miR-1307-5p suppresses proliferation and tumorigenesis of bladder cancer via targeting MDM4 and the Hippo signaling pathway
Source: Discov Oncol. 2022 Jul 1;13:57. doi: 10.1007/s12672-022-00512-2 (PMC9249964; doi:10.1007/s12672-022-00512-2)

# GO:ALL Enrichment

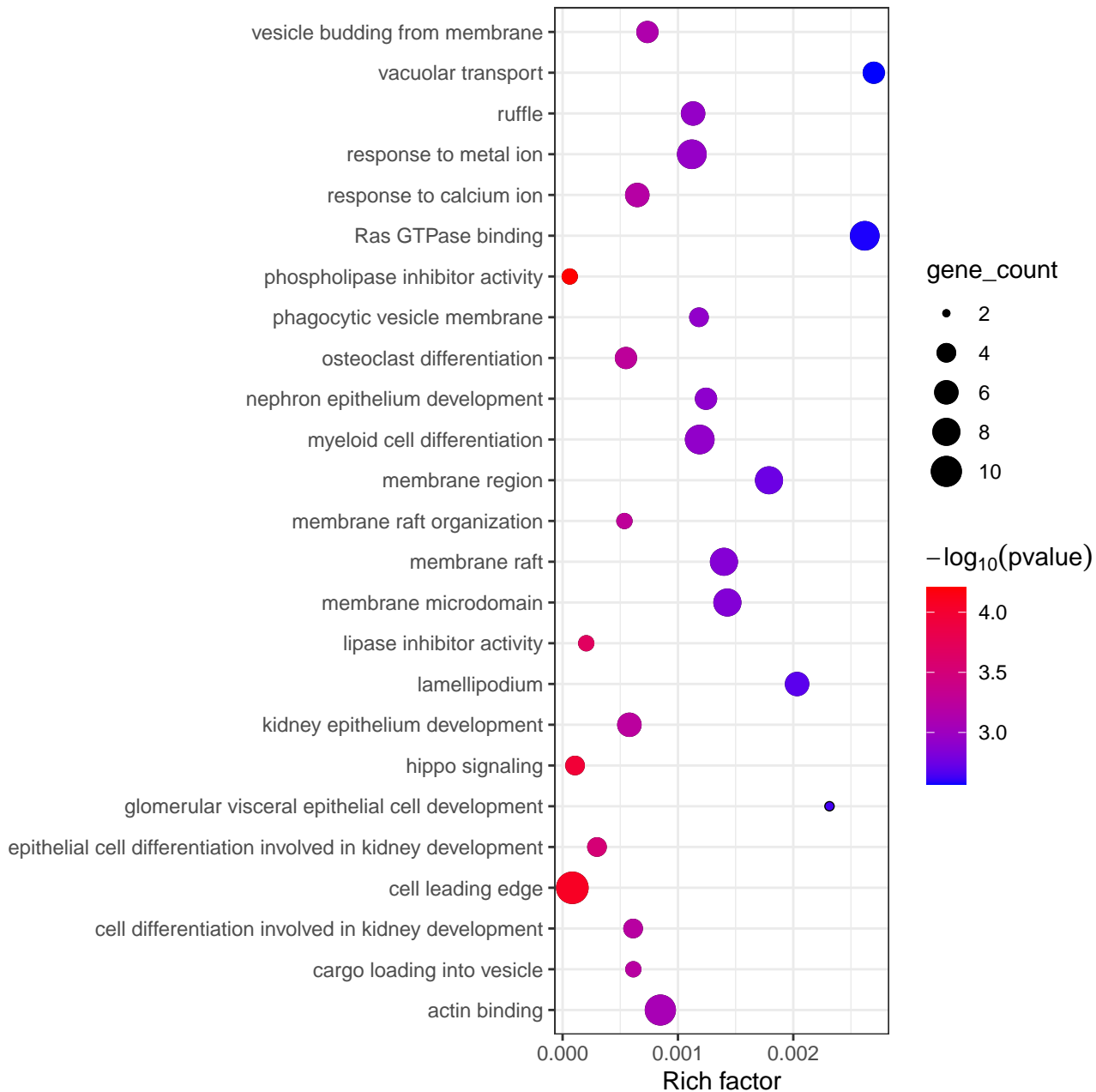

Supplement: Supplementary file 1 — Additional file 1: Figure S1A. Bubble plot of miR-1307-5p from the GO enrichment analysis. (PDF 8 KB) [file 12672_2022_512_MOESM1_ESM.pdf]

# KEGG Enrichment

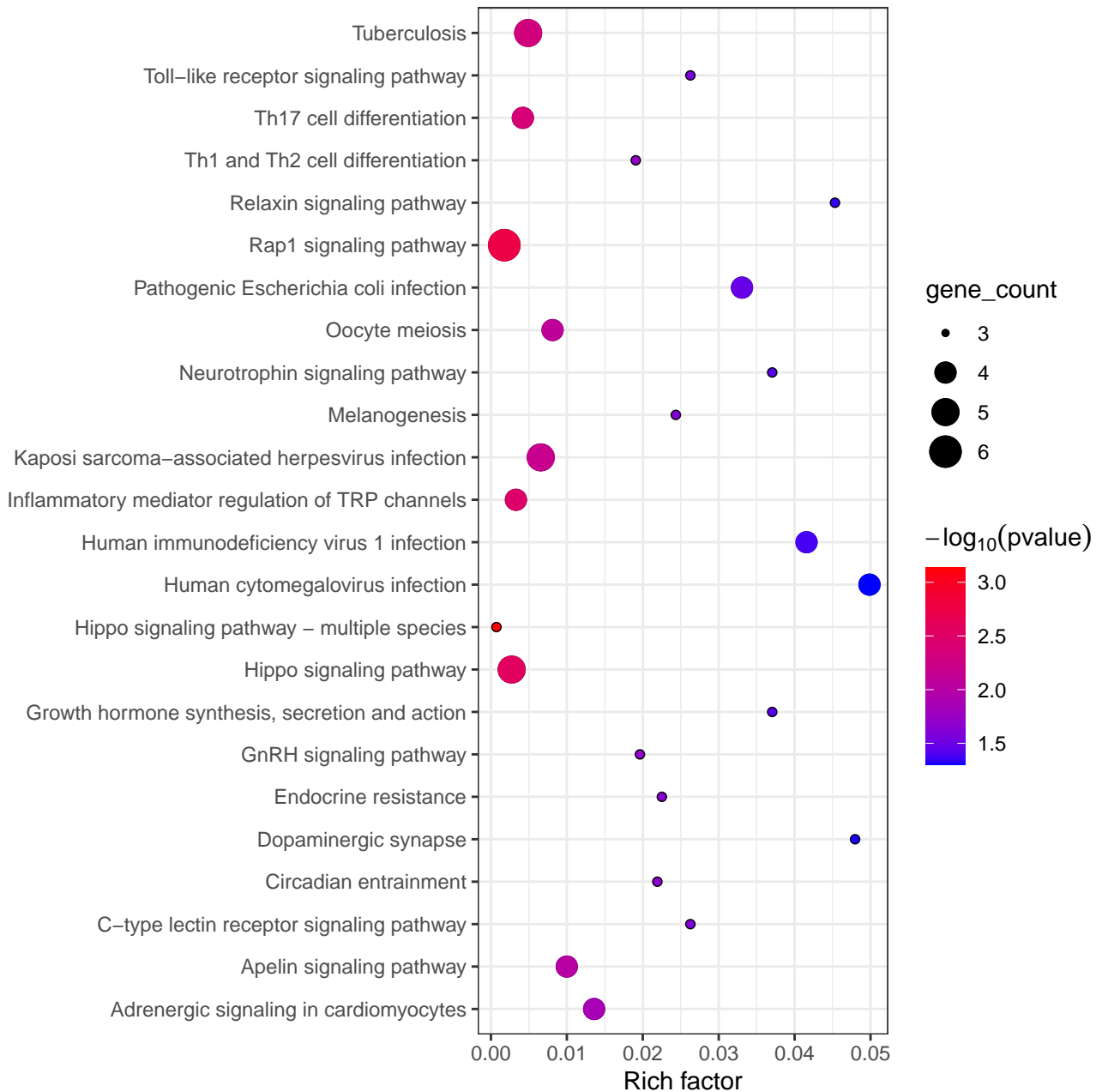

Supplement: Supplementary file 2 — Additional file 2: Figure S1B. Bubble plot of miR-1307-5p from the KEGG enrichment analysis (PDF 8 KB) [file 12672_2022_512_MOESM2_ESM.pdf]
